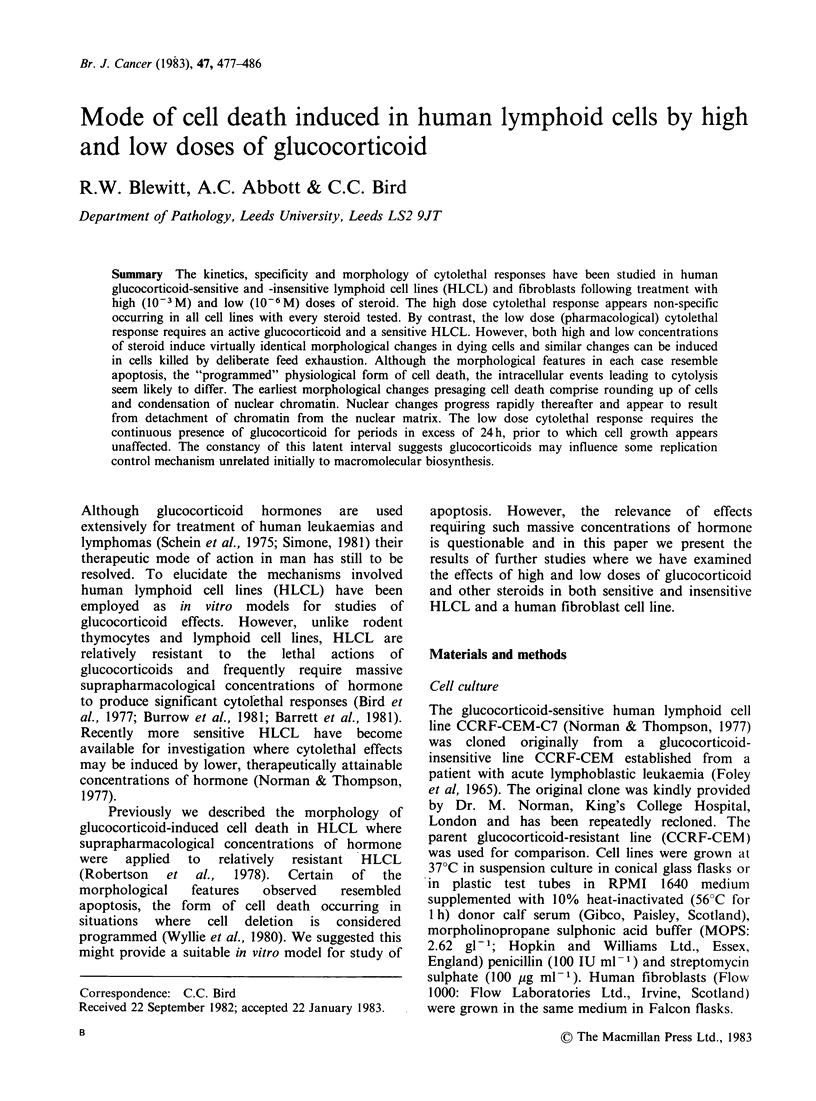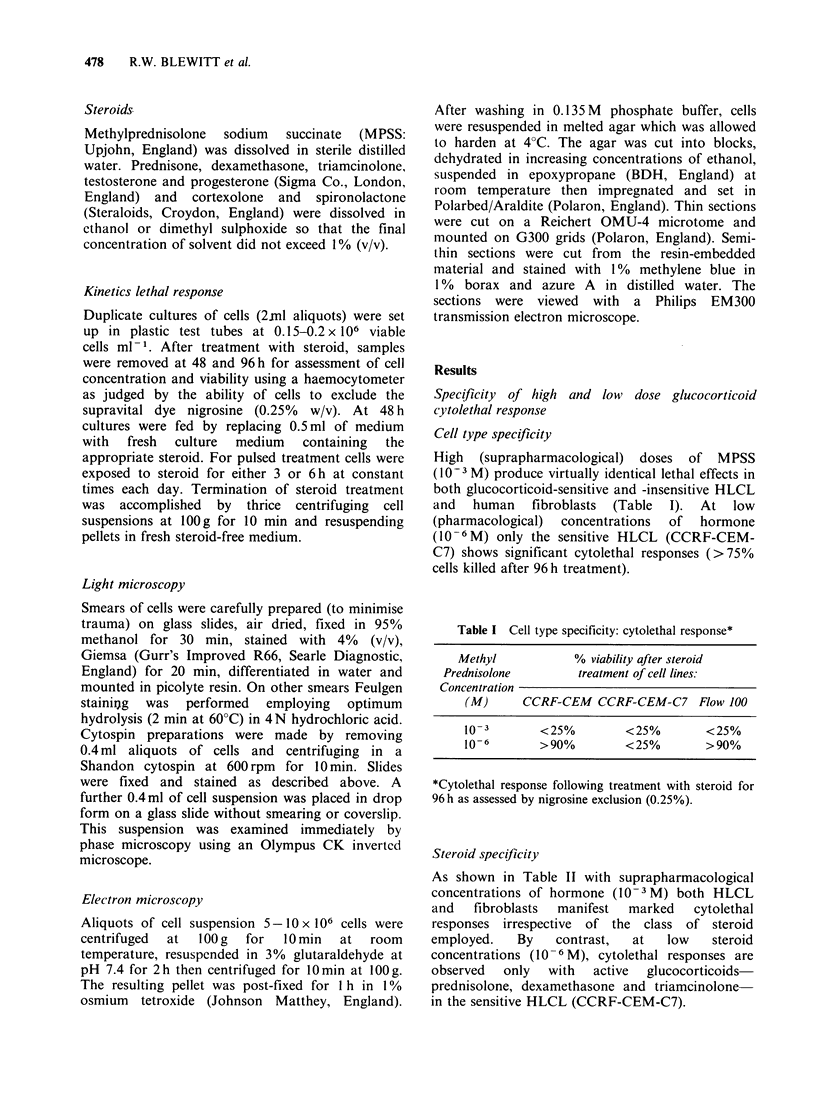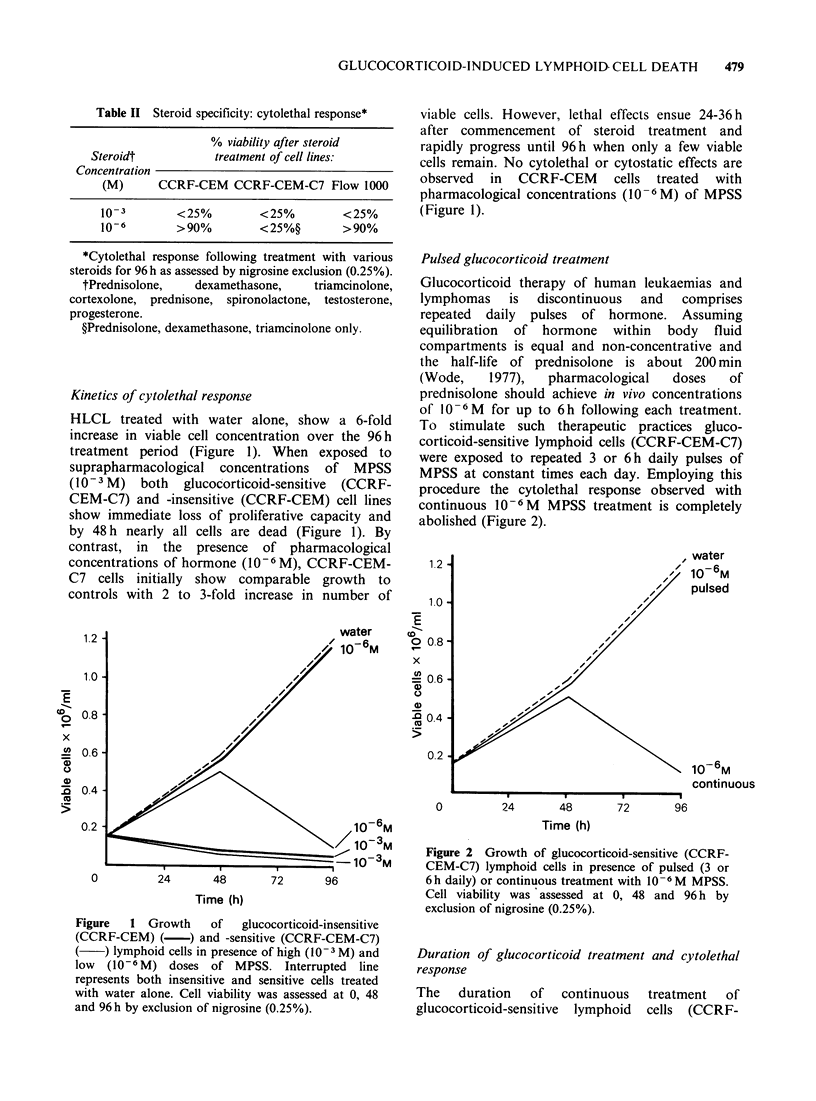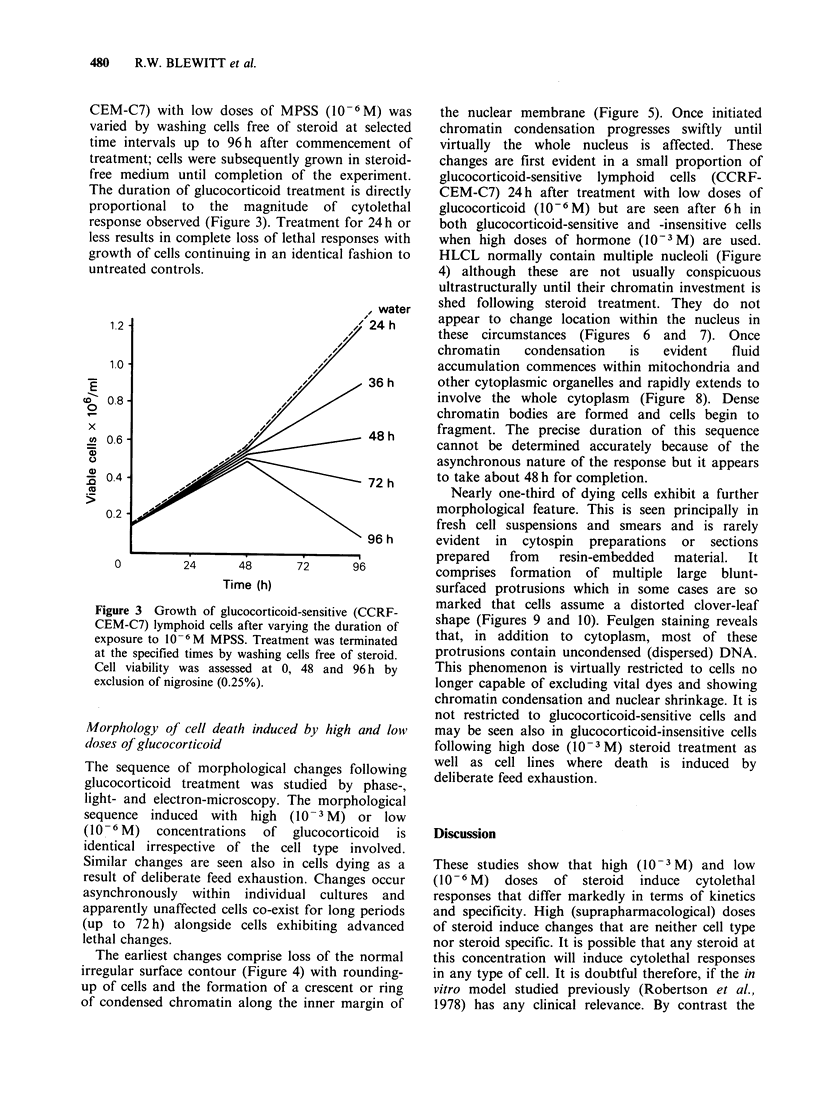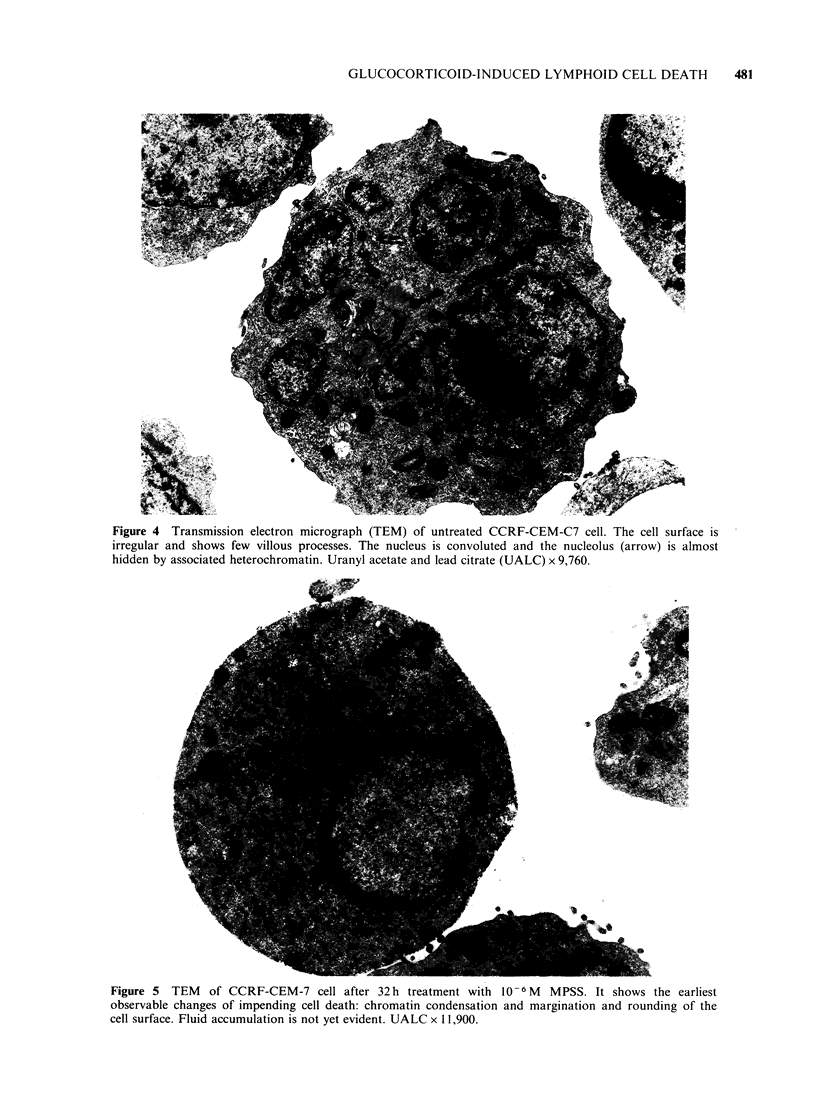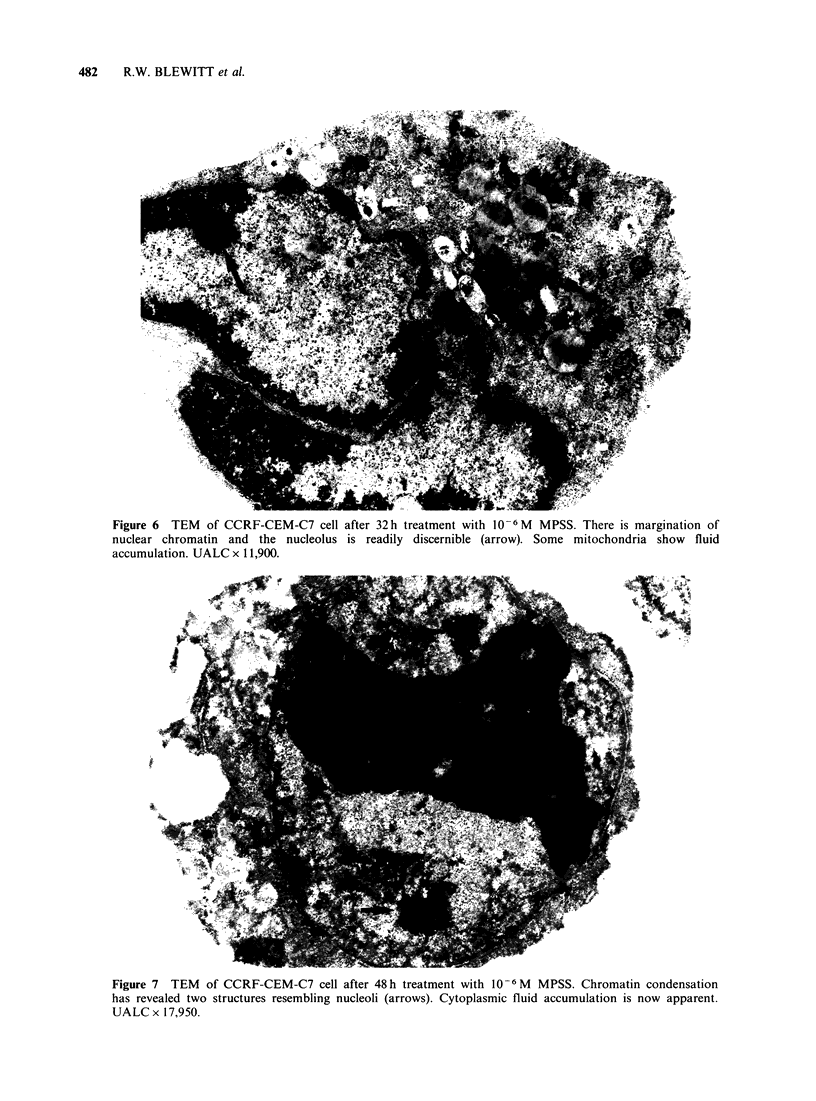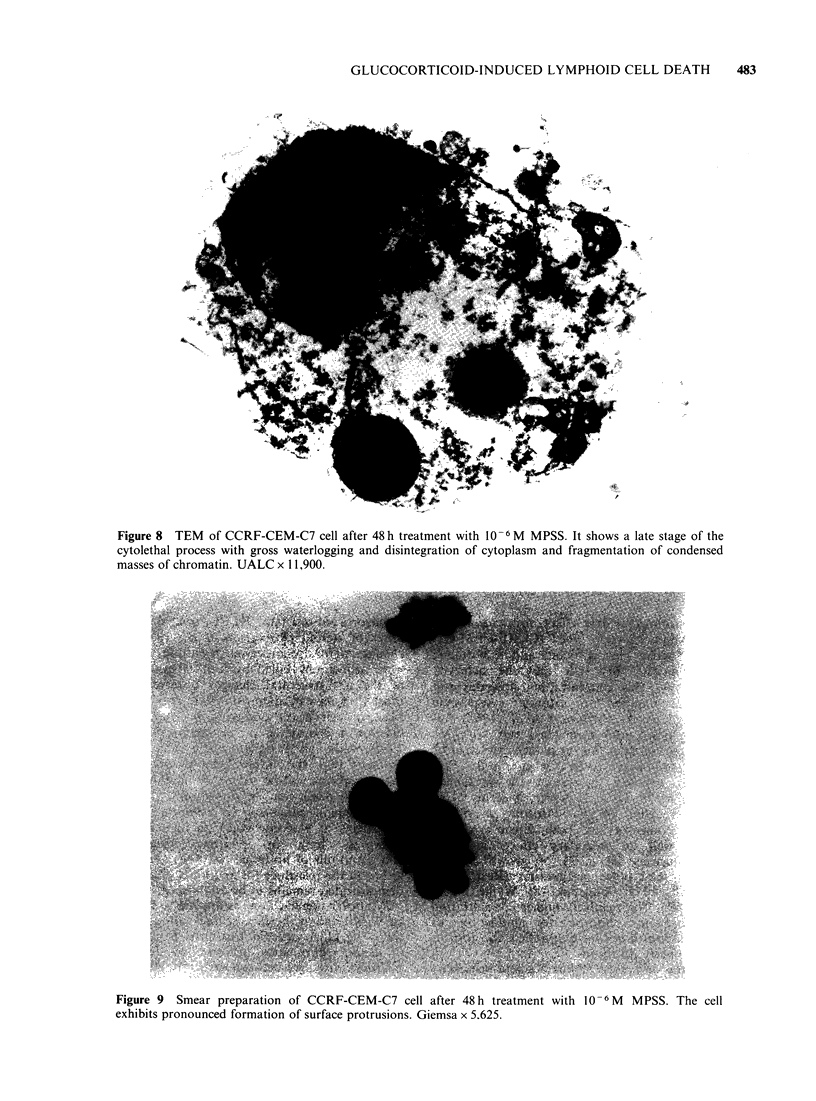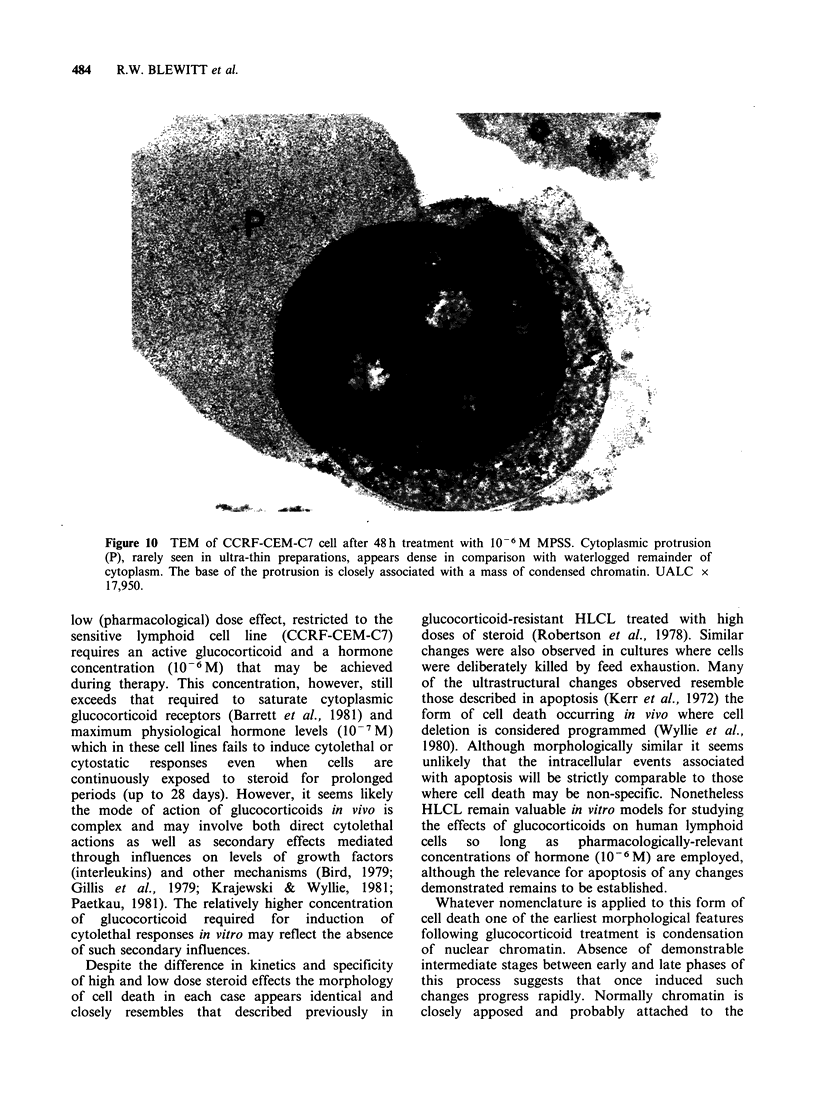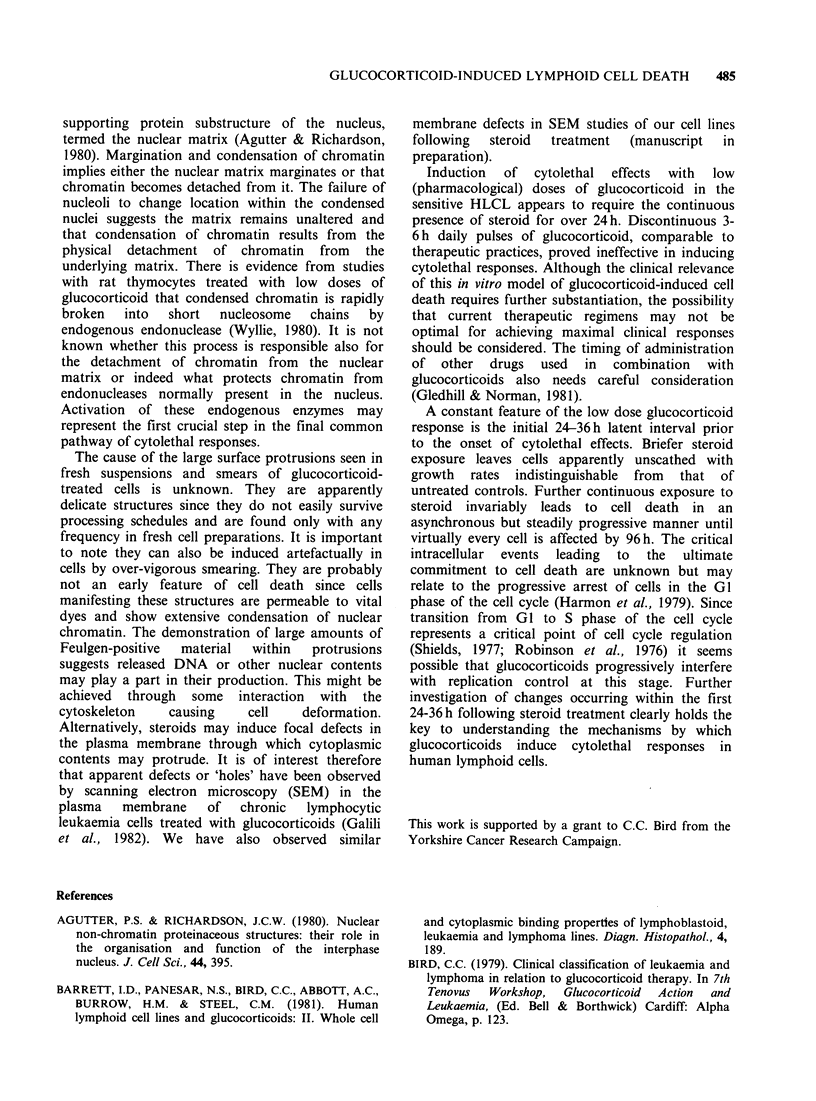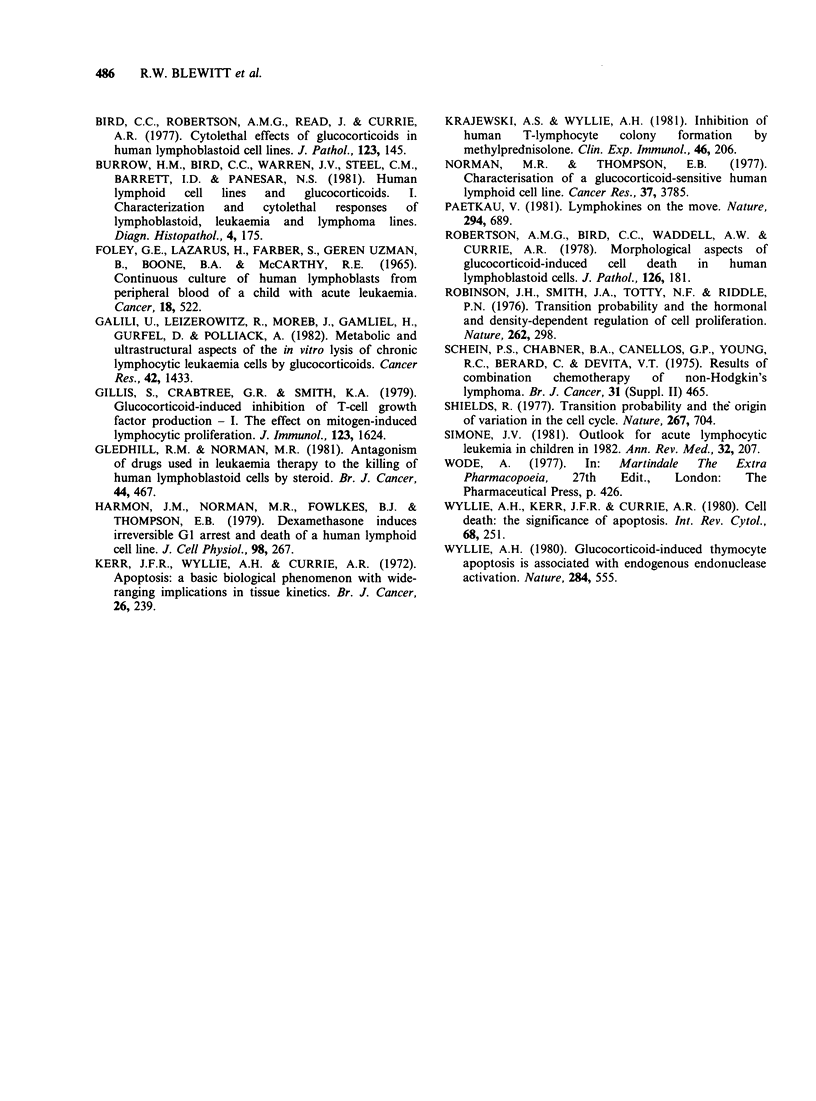# Mode of cell death induced in human lymphoid cells by high and low doses of glucocorticoid.

**DOI:** 10.1038/bjc.1983.77

**Published:** 1983-04

**Authors:** R. W. Blewitt, A. C. Abbott, C. C. Bird

## Abstract

**Images:**


*Br. J. Cancer* (1983), **47**, 477–486

### Mode of cell death induced in human lymphoid cells by high and low doses of glucocorticoid

R.W. Blewitt, A.C. Abbott & C.C. Bird

*Department of Pathology, Leeds University, Leeds LS2 9JT*

**Summary** The kinetics, specificity and morphology of cytolethal responses have been studied in human glucocorticoid-sensitive and -insensitive lymphoid cell lines (HLCL) and fibroblasts following treatment with high ($10^{-3}$M) and low ($10^{-6}$M) doses of steroid. The high dose cytolethal response appears non-specific occurring in all cell lines with every steroid tested. By contrast, the low dose (pharmacological) cytolethal response requires an active glucocorticoid and a sensitive HLCL. However, both high and low concentrations of steroid induce virtually identical morphological changes in dying cells and similar changes can be induced in cells killed by deliberate feed exhaustion. Although the morphological features in each case resemble apoptosis, the "programmed" physiological form of cell death, the intracellular events leading to cytolysis seem likely to differ. The earliest morphological changes presaging cell death comprise rounding up of cells and condensation of nuclear chromatin. Nuclear changes progress rapidly thereafter and appear to result from detachment of chromatin from the nuclear matrix. The low dose cytolethal response requires the continuous presence of glucocorticoid for periods in excess of 24 h, prior to which cell growth appears unaffected. The constancy of this latent interval suggests glucocorticoids may influence some replication control mechanism unrelated initially to macromolecular biosynthesis.

Although glucocorticoid hormones are used extensively for treatment of human leukaemias and lymphomas (Schein *et al.*, 1975; Simone, 1981) their therapeutic mode of action in man has still to be resolved. To elucidate the mechanisms involved human lymphoid cell lines (HLCL) have been employed as *in vitro* models for studies of glucocorticoid effects. However, unlike rodent thymocytes and lymphoid cell lines, HLCL are relatively resistant to the lethal actions of glucocorticoids and frequently require massive suprapharmacological concentrations of hormone to produce significant cytolethal responses (Bird *et al.*, 1977; Burrow *et al.*, 1981; Barrett *et al.*, 1981). Recently more sensitive HLCL have become available for investigation where cytolethal effects may be induced by lower, therapeutically attainable concentrations of hormone (Norman & Thompson, 1977).

Previously we described the morphology of glucocorticoid-induced cell death in HLCL where suprapharmacological concentrations of hormone were applied to relatively resistant HLCL (Robertson *et al.*, 1978). Certain of the morphological features observed resembled apoptosis, the form of cell death occurring in situations where cell deletion is considered programmed (Wyllie *et al.*, 1980). We suggested this might provide a suitable *in vitro* model for study of apoptosis. However, the relevance of effects requiring such massive concentrations of hormone is questionable and in this paper we present the results of further studies where we have examined the effects of high and low doses of glucocorticoid and other steroids in both sensitive and insensitive HLCL and a human fibroblast cell line.

### Materials and methods

### *Cell culture*

The glucocorticoid-sensitive human lymphoid cell line CCRF-CEM-C7 (Norman & Thompson, 1977) was cloned originally from a glucocorticoid-insensitive line CCRF-CEM established from a patient with acute lymphoblastic leukaemia (Foley *et al*, 1965). The original clone was kindly provided by Dr. M. Norman, King's College Hospital, London and has been repeatedly recloned. The parent glucocorticoid-resistant line (CCRF-CEM) was used for comparison. Cell lines were grown at 37°C in suspension culture in conical glass flasks or in plastic test tubes in RPMI 1640 medium supplemented with 10% heat-inactivated (56°C for 1 h) donor calf serum (Gibco, Paisley, Scotland), morpholinopropane sulphonic acid buffer (MOPS: 2.62 $gl^{-1}$; Hopkin and Williams Ltd., Essex, England) penicillin (100 IU $ml^{-1}$) and streptomycin sulphate (100 $\mu g$ $ml^{-1}$). Human fibroblasts (Flow 1000: Flow Laboratories Ltd., Irvine, Scotland) were grown in the same medium in Falcon flasks.

Correspondence: C.C. Bird
Received 22 September 1982; accepted 22 January 1983.

© The Macmillan Press Ltd., 1983

*Steroids*

Methylprednisolone sodium succinate (MPSS: Upjohn, England) was dissolved in sterile distilled water. Prednisone, dexamethasone, triamcinolone, testosterone and progesterone (Sigma Co., London, England) and cortexolone and spironolactone (Steraloids, Croydon, England) were dissolved in ethanol or dimethyl sulphoxide so that the final concentration of solvent did not exceed 1% (v/v).

*Kinetics lethal response*

Duplicate cultures of cells (2ml aliquots) were set up in plastic test tubes at $0.15–0.2 \times 10^6$ viable cells $ml^{-1}$. After treatment with steroid, samples were removed at 48 and 96 h for assessment of cell concentration and viability using a haemocytometer as judged by the ability of cells to exclude the supravital dye nigrosine (0.25% w/v). At 48 h cultures were fed by replacing 0.5 ml of medium with fresh culture medium containing the appropriate steroid. For pulsed treatment cells were exposed to steroid for either 3 or 6 h at constant times each day. Termination of steroid treatment was accomplished by thrice centrifuging cell suspensions at 100 g for 10 min and resuspending pellets in fresh steroid-free medium.

*Light microscopy*

Smears of cells were carefully prepared (to minimise trauma) on glass slides, air dried, fixed in 95% methanol for 30 min, stained with 4% (v/v), Giemsa (Gurr's Improved R66, Searle Diagnostic, England) for 20 min, differentiated in water and mounted in picolyte resin. On other smears Feulgen staining was performed employing optimum hydrolysis (2 min at 60°C) in 4 N hydrochloric acid. Cytospin preparations were made by removing 0.4 ml aliquots of cells and centrifuging in a Shandon cytospin at 600 rpm for 10 min. Slides were fixed and stained as described above. A further 0.4 ml of cell suspension was placed in drop form on a glass slide without smearing or coverslip. This suspension was examined immediately by phase microscopy using an Olympus CK inverted microscope.

*Electron microscopy*

Aliquots of cell suspension $5-10 \times 10^6$ cells were centrifuged at 100 g for 10 min at room temperature, resuspended in 3% glutaraldehyde at pH 7.4 for 2 h then centrifuged for 10 min at 100 g. The resulting pellet was post-fixed for 1 h in 1% osmium tetroxide (Johnson Matthey, England). After washing in 0.135 M phosphate buffer, cells were resuspended in melted agar which was allowed to harden at 4°C. The agar was cut into blocks, dehydrated in increasing concentrations of ethanol, suspended in epoxypropane (BDH, England) at room temperature then impregnated and set in Polarbed/Araldite (Polaron, England). Thin sections were cut on a Reichert OMU-4 microtome and mounted on G300 grids (Polaron, England). Semi-thin sections were cut from the resin-embedded material and stained with 1% methylene blue in 1% borax and azure A in distilled water. The sections were viewed with a Philips EM300 transmission electron microscope.

### Results

*Specificity of high and low dose glucocorticoid cytolethal response*

*Cell type specificity*

High (suprapharmacological) doses of MPSS ($10^{-3}$ M) produce virtually identical lethal effects in both glucocorticoid-sensitive and -insensitive HLCL and human fibroblasts (Table I). At low (pharmacological) concentrations of hormone ($10^{-6}$ M) only the sensitive HLCL (CCRF-CEM-C7) shows significant cytolethal responses (>75% cells killed after 96 h treatment).

**Table I** Cell type specificity: cytolethal response*

| *Methyl Prednisolone Concentration (M)* | *% viability after steroid treatment of cell lines:* | | |
|---|---|---|---|
| | *CCRF-CEM* | *CCRF-CEM-C7* | *Flow 100* |
| $10^{-3}$ | <25% | <25% | <25% |
| $10^{-6}$ | >90% | <25% | >90% |

*Cytolethal response following treatment with steroid for 96 h as assessed by nigrosine exclusion (0.25%).

*Steroid specificity*

As shown in Table II with suprapharmacological concentrations of hormone ($10^{-3}$ M) both HLCL and fibroblasts manifest marked cytolethal responses irrespective of the class of steroid employed. By contrast, at low steroid concentrations ($10^{-6}$ M), cytolethal responses are observed only with active glucocorticoids—prednisolone, dexamethasone and triamcinolone—in the sensitive HLCL (CCRF-CEM-C7).

**Table II** Steroid specificity: cytolethal response*

| *Steroid*† *Concentration* (M) | *% viability after steroid treatment of cell lines:* CCRF-CEM | CCRF-CEM-C7 | Flow 1000 |
|---|---|---|---|
| $10^{-3}$ | <25% | <25% | <25% |
| $10^{-6}$ | >90% | <25%§ | >90% |

*Cytolethal response following treatment with various steroids for 96 h as assessed by nigrosine exclusion (0.25%).

†Prednisolone, dexamethasone, triamcinolone, cortexolone, prednisone, spironolactone, testosterone, progesterone.

§Prednisolone, dexamethasone, triamcinolone only.

### *Kinetics of cytolethal response*

HLCL treated with water alone, show a 6-fold increase in viable cell concentration over the 96 h treatment period (Figure 1). When exposed to suprapharmacological concentrations of MPSS ($10^{-3}$ M) both glucocorticoid-sensitive (CCRF-CEM-C7) and -insensitive (CCRF-CEM) cell lines show immediate loss of proliferative capacity and by 48 h nearly all cells are dead (Figure 1). By contrast, in the presence of pharmacological concentrations of hormone ($10^{-6}$ M), CCRF-CEM-C7 cells initially show comparable growth to controls with 2 to 3-fold increase in number of viable cells. However, lethal effects ensue 24-36 h after commencement of steroid treatment and rapidly progress until 96 h when only a few viable cells remain. No cytolethal or cytostatic effects are observed in CCRF-CEM cells treated with pharmacological concentrations ($10^{-6}$ M) of MPSS (Figure 1).

**Figure 1** Growth of glucocorticoid-insensitive (CCRF-CEM) (——) and -sensitive (CCRF-CEM-C7) (——) lymphoid cells in presence of high ($10^{-3}$ M) and low ($10^{-6}$ M) doses of MPSS. Interrupted line represents both insensitive and sensitive cells treated with water alone. Cell viability was assessed at 0, 48 and 96 h by exclusion of nigrosine (0.25%).

### *Pulsed glucocorticoid treatment*

Glucocorticoid therapy of human leukaemias and lymphomas is discontinuous and comprises repeated daily pulses of hormone. Assuming equilibration of hormone within body fluid compartments is equal and non-concentrative and the half-life of prednisolone is about 200 min (Wode, 1977), pharmacological doses of prednisolone should achieve *in vivo* concentrations of $10^{-6}$ M for up to 6 h following each treatment. To stimulate such therapeutic practices glucocorticoid-sensitive lymphoid cells (CCRF-CEM-C7) were exposed to repeated 3 or 6 h daily pulses of MPSS at constant times each day. Employing this procedure the cytolethal response observed with continuous $10^{-6}$ M MPSS treatment is completely abolished (Figure 2).

**Figure 2** Growth of glucocorticoid-sensitive (CCRF-CEM-C7) lymphoid cells in presence of pulsed (3 or 6 h daily) or continuous treatment with $10^{-6}$ M MPSS. Cell viability was assessed at 0, 48 and 96 h by exclusion of nigrosine (0.25%).

### *Duration of glucocorticoid treatment and cytolethal response*

The duration of continuous treatment of glucocorticoid-sensitive lymphoid cells (CCRF-

CEM-C7) with low doses of MPSS ($10^{-6}$ M) was varied by washing cells free of steroid at selected time intervals up to 96 h after commencement of treatment; cells were subsequently grown in steroid-free medium until completion of the experiment. The duration of glucocorticoid treatment is directly proportional to the magnitude of cytolethal response observed (Figure 3). Treatment for 24 h or less results in complete loss of lethal responses with growth of cells continuing in an identical fashion to untreated controls.

**Figure 3** Growth of glucocorticoid-sensitive (CCRF-CEM-C7) lymphoid cells after varying the duration of exposure to $10^{-6}$ M MPSS. Treatment was terminated at the specified times by washing cells free of steroid. Cell viability was assessed at 0, 48 and 96 h by exclusion of nigrosine (0.25%).

### *Morphology of cell death induced by high and low doses of glucocorticoid*

The sequence of morphological changes following glucocorticoid treatment was studied by phase-, light- and electron-microscopy. The morphological sequence induced with high ($10^{-3}$ M) or low ($10^{-6}$ M) concentrations of glucocorticoid is identical irrespective of the cell type involved. Similar changes are seen also in cells dying as a result of deliberate feed exhaustion. Changes occur asynchronously within individual cultures and apparently unaffected cells co-exist for long periods (up to 72 h) alongside cells exhibiting advanced lethal changes.

The earliest changes comprise loss of the normal irregular surface contour (Figure 4) with rounding-up of cells and the formation of a crescent or ring of condensed chromatin along the inner margin of the nuclear membrane (Figure 5). Once initiated chromatin condensation progresses swiftly until virtually the whole nucleus is affected. These changes are first evident in a small proportion of glucocorticoid-sensitive lymphoid cells (CCRF-CEM-C7) 24 h after treatment with low doses of glucocorticoid ($10^{-6}$ M) but are seen after 6 h in both glucocorticoid-sensitive and -insensitive cells when high doses of hormone ($10^{-3}$ M) are used. HLCL normally contain multiple nucleoli (Figure 4) although these are not usually conspicuous ultrastructurally until their chromatin investment is shed following steroid treatment. They do not appear to change location within the nucleus in these circumstances (Figures 6 and 7). Once chromatin condensation is evident fluid accumulation commences within mitochondria and other cytoplasmic organelles and rapidly extends to involve the whole cytoplasm (Figure 8). Dense chromatin bodies are formed and cells begin to fragment. The precise duration of this sequence cannot be determined accurately because of the asynchronous nature of the response but it appears to take about 48 h for completion.

Nearly one-third of dying cells exhibit a further morphological feature. This is seen principally in fresh cell suspensions and smears and is rarely evident in cytospin preparations or sections prepared from resin-embedded material. It comprises formation of multiple large blunt-surfaced protrusions which in some cases are so marked that cells assume a distorted clover-leaf shape (Figures 9 and 10). Feulgen staining reveals that, in addition to cytoplasm, most of these protrusions contain uncondensed (dispersed) DNA. This phenomenon is virtually restricted to cells no longer capable of excluding vital dyes and showing chromatin condensation and nuclear shrinkage. It is not restricted to glucocorticoid-sensitive cells and may be seen also in glucocorticoid-insensitive cells following high dose ($10^{-3}$ M) steroid treatment as well as cell lines where death is induced by deliberate feed exhaustion.

### Discussion

These studies show that high ($10^{-3}$ M) and low ($10^{-6}$ M) doses of steroid induce cytolethal responses that differ markedly in terms of kinetics and specificity. High (suprapharmacological) doses of steroid induce changes that are neither cell type nor steroid specific. It is possible that any steroid at this concentration will induce cytolethal responses in any type of cell. It is doubtful therefore, if the *in vitro* model studied previously (Robertson *et al.*, 1978) has any clinical relevance. By contrast the

**Figure 4** Transmission electron micrograph (TEM) of untreated CCRF-CEM-C7 cell. The cell surface is irregular and shows few villous processes. The nucleus is convoluted and the nucleolus (arrow) is almost hidden by associated heterochromatin. Uranyl acetate and lead citrate (UALC) × 9,760.

**Figure 5** TEM of CCRF-CEM-7 cell after 32 h treatment with $10^{-6}$ M MPSS. It shows the earliest observable changes of impending cell death: chromatin condensation and margination and rounding of the cell surface. Fluid accumulation is not yet evident. UALC × 11,900.

**Figure 6** TEM of CCRF-CEM-C7 cell after 32 h treatment with $10^{-6}$ M MPSS. There is margination of nuclear chromatin and the nucleolus is readily discernible (arrow). Some mitochondria show fluid accumulation. UALC × 11,900.

**Figure 7** TEM of CCRF-CEM-C7 cell after 48 h treatment with $10^{-6}$ M MPSS. Chromatin condensation has revealed two structures resembling nucleoli (arrows). Cytoplasmic fluid accumulation is now apparent. UALC × 17,950.

**Figure 8** TEM of CCRF-CEM-C7 cell after 48 h treatment with $10^{-6}$ M MPSS. It shows a late stage of the cytolethal process with gross waterlogging and disintegration of cytoplasm and fragmentation of condensed masses of chromatin. UALC × 11,900.

**Figure 9** Smear preparation of CCRF-CEM-C7 cell after 48 h treatment with $10^{-6}$ M MPSS. The cell exhibits pronounced formation of surface protrusions. Giemsa × 5.625.

**Figure 10** TEM of CCRF-CEM-C7 cell after 48 h treatment with $10^{-6}$ M MPSS. Cytoplasmic protrusion (P), rarely seen in ultra-thin preparations, appears dense in comparison with waterlogged remainder of cytoplasm. The base of the protrusion is closely associated with a mass of condensed chromatin. UALC × 17,950.

low (pharmacological) dose effect, restricted to the sensitive lymphoid cell line (CCRF-CEM-C7) requires an active glucocorticoid and a hormone concentration ($10^{-6}$ M) that may be achieved during therapy. This concentration, however, still exceeds that required to saturate cytoplasmic glucocorticoid receptors (Barrett *et al.*, 1981) and maximum physiological hormone levels ($10^{-7}$ M) which in these cell lines fails to induce cytolethal or cytostatic responses even when cells are continuously exposed to steroid for prolonged periods (up to 28 days). However, it seems likely the mode of action of glucocorticoids *in vivo* is complex and may involve both direct cytolethal actions as well as secondary effects mediated through influences on levels of growth factors (interleukins) and other mechanisms (Bird, 1979; Gillis *et al.*, 1979; Krajewski & Wyllie, 1981; Paetkau, 1981). The relatively higher concentration of glucocorticoid required for induction of cytolethal responses *in vitro* may reflect the absence of such secondary influences.

Despite the difference in kinetics and specificity of high and low dose steroid effects the morphology of cell death in each case appears identical and closely resembles that described previously in glucocorticoid-resistant HLCL treated with high doses of steroid (Robertson *et al.*, 1978). Similar changes were also observed in cultures where cells were deliberately killed by feed exhaustion. Many of the ultrastructural changes observed resemble those described in apoptosis (Kerr *et al.*, 1972) the form of cell death occurring *in vivo* where cell deletion is considered programmed (Wyllie *et al.*, 1980). Although morphologically similar it seems unlikely that the intracellular events associated with apoptosis will be strictly comparable to those where cell death may be non-specific. Nonetheless HLCL remain valuable *in vitro* models for studying the effects of glucocorticoids on human lymphoid cells so long as pharmacologically-relevant concentrations of hormone ($10^{-6}$ M) are employed, although the relevance for apoptosis of any changes demonstrated remains to be established.

Whatever nomenclature is applied to this form of cell death one of the earliest morphological features following glucocorticoid treatment is condensation of nuclear chromatin. Absence of demonstrable intermediate stages between early and late phases of this process suggests that once induced such changes progress rapidly. Normally chromatin is closely apposed and probably attached to the supporting protein substructure of the nucleus, termed the nuclear matrix (Agutter & Richardson, 1980). Margination and condensation of chromatin implies either the nuclear matrix marginates or that chromatin becomes detached from it. The failure of nucleoli to change location within the condensed nuclei suggests the matrix remains unaltered and that condensation of chromatin results from the physical detachment of chromatin from the underlying matrix. There is evidence from studies with rat thymocytes treated with low doses of glucocorticoid that condensed chromatin is rapidly broken into short nucleosome chains by endogenous endonuclease (Wyllie, 1980). It is not known whether this process is responsible also for the detachment of chromatin from the nuclear matrix or indeed what protects chromatin from endonucleases normally present in the nucleus. Activation of these endogenous enzymes may represent the first crucial step in the final common pathway of cytolethal responses.

The cause of the large surface protrusions seen in fresh suspensions and smears of glucocorticoid-treated cells is unknown. They are apparently delicate structures since they do not easily survive processing schedules and are found only with any frequency in fresh cell preparations. It is important to note they can also be induced artefactually in cells by over-vigorous smearing. They are probably not an early feature of cell death since cells manifesting these structures are permeable to vital dyes and show extensive condensation of nuclear chromatin. The demonstration of large amounts of Feulgen-positive material within protrusions suggests released DNA or other nuclear contents may play a part in their production. This might be achieved through some interaction with the cytoskeleton causing cell deformation. Alternatively, steroids may induce focal defects in the plasma membrane through which cytoplasmic contents may protrude. It is of interest therefore that apparent defects or 'holes' have been observed by scanning electron microscopy (SEM) in the plasma membrane of chronic lymphocytic leukaemia cells treated with glucocorticoids (Galili *et al.*, 1982). We have also observed similar membrane defects in SEM studies of our cell lines following steroid treatment (manuscript in preparation).

Induction of cytolethal effects with low (pharmacological) doses of glucocorticoid in the sensitive HLCL appears to require the continuous presence of steroid for over 24 h. Discontinuous 3-6 h daily pulses of glucocorticoid, comparable to therapeutic practices, proved ineffective in inducing cytolethal responses. Although the clinical relevance of this *in vitro* model of glucocorticoid-induced cell death requires further substantiation, the possibility that current therapeutic regimens may not be optimal for achieving maximal clinical responses should be considered. The timing of administration of other drugs used in combination with glucocorticoids also needs careful consideration (Gledhill & Norman, 1981).

A constant feature of the low dose glucocorticoid response is the initial 24–36 h latent interval prior to the onset of cytolethal effects. Briefer steroid exposure leaves cells apparently unscathed with growth rates indistinguishable from that of untreated controls. Further continuous exposure to steroid invariably leads to cell death in an asynchronous but steadily progressive manner until virtually every cell is affected by 96 h. The critical intracellular events leading to the ultimate commitment to cell death are unknown but may relate to the progressive arrest of cells in the G1 phase of the cell cycle (Harmon *et al.*, 1979). Since transition from G1 to S phase of the cell cycle represents a critical point of cell cycle regulation (Shields, 1977; Robinson *et al.*, 1976) it seems possible that glucocorticoids progressively interfere with replication control at this stage. Further investigation of changes occurring within the first 24-36 h following steroid treatment clearly holds the key to understanding the mechanisms by which glucocorticoids induce cytolethal responses in human lymphoid cells.

This work is supported by a grant to C.C. Bird from the Yorkshire Cancer Research Campaign.

### References

AGUTTER, P.S. & RICHARDSON, J.C.W. (1980). Nuclear non-chromatin proteinaceous structures: their role in the organisation and function of the interphase nucleus. *J. Cell Sci.*, **44**, 395.

BARRETT, I.D., PANESAR, N.S., BIRD, C.C., ABBOTT, A.C., BURROW, H.M. & STEEL, C.M. (1981). Human lymphoid cell lines and glucocorticoids: II. Whole cell and cytoplasmic binding properties of lymphoblastoid, leukaemia and lymphoma lines. *Diagn. Histopathol.*, **4**, 189.

BIRD, C.C. (1979). Clinical classification of leukaemia and lymphoma in relation to glucocorticoid therapy. In *7th Tenovus Workshop, Glucocorticoid Action and Leukaemia*, (Ed. Bell & Borthwick) Cardiff: Alpha Omega, p. 123.

BIRD, C.C., ROBERTSON, A.M.G., READ, J. & CURRIE, A.R. (1977). Cytolethal effects of glucocorticoids in human lymphoblastoid cell lines. *J. Pathol.*, **123**, 145.

BURROW, H.M., BIRD, C.C., WARREN, J.V., STEEL, C.M., BARRETT, I.D. & PANESAR, N.S. (1981). Human lymphoid cell lines and glucocorticoids. I. Characterization and cytolethal responses of lymphoblastoid, leukaemia and lymphoma lines. *Diagn. Histopathol.*, **4**, 175.

FOLEY, G.E., LAZARUS, H., FARBER, S., GEREN UZMAN, B., BOONE, B.A. & McCARTHY, R.E. (1965). Continuous culture of human lymphoblasts from peripheral blood of a child with acute leukaemia. *Cancer*, **18**, 522.

GALILI, U., LEIZEROWITZ, R., MOREB, J., GAMLIEL, H., GURFEL, D. & POLLIACK, A. (1982). Metabolic and ultrastructural aspects of the *in vitro* lysis of chronic lymphocytic leukaemia cells by glucocorticoids. *Cancer Res.*, **42**, 1433.

GILLIS, S., CRABTREE, G.R. & SMITH, K.A. (1979). Glucocorticoid-induced inhibition of T-cell growth factor production – I. The effect on mitogen-induced lymphocytic proliferation. *J. Immunol.*, **123**, 1624.

GLEDHILL, R.M. & NORMAN, M.R. (1981). Antagonism of drugs used in leukaemia therapy to the killing of human lymphoblastoid cells by steroid. *Br. J. Cancer*, **44**, 467.

HARMON, J.M., NORMAN, M.R., FOWLKES, B.J. & THOMPSON, E.B. (1979). Dexamethasone induces irreversible G1 arrest and death of a human lymphoid cell line. *J. Cell Physiol.*, **98**, 267.

KERR, J.F.R., WYLLIE, A.H. & CURRIE, A.R. (1972). Apoptosis: a basic biological phenomenon with wide-ranging implications in tissue kinetics. *Br. J. Cancer*, **26**, 239.

KRAJEWSKI, A.S. & WYLLIE, A.H. (1981). Inhibition of human T-lymphocyte colony formation by methylprednisolone. *Clin. Exp. Immunol.*, **46**, 206.

NORMAN, M.R. & THOMPSON, E.B. (1977). Characterisation of a glucocorticoid-sensitive human lymphoid cell line. *Cancer Res.*, **37**, 3785.

PAETKAU, V. (1981). Lymphokines on the move. *Nature*, **294**, 689.

ROBERTSON, A.M.G., BIRD, C.C., WADDELL, A.W. & CURRIE, A.R. (1978). Morphological aspects of glucocorticoid-induced cell death in human lymphoblastoid cells. *J. Pathol.*, **126**, 181.

ROBINSON, J.H., SMITH, J.A., TOTTY, N.F. & RIDDLE, P.N. (1976). Transition probability and the hormonal and density-dependent regulation of cell proliferation. *Nature*, **262**, 298.

SCHEIN, P.S., CHABNER, B.A., CANELLOS, G.P., YOUNG, R.C., BERARD, C. & DEVITA, V.T. (1975). Results of combination chemotherapy of non-Hodgkin's lymphoma. *Br. J. Cancer*, **31** (Suppl. II) 465.

SHIELDS, R. (1977). Transition probability and the origin of variation in the cell cycle. *Nature*, **267**, 704.

SIMONE, J.V. (1981). Outlook for acute lymphocytic leukemia in children in 1982. *Ann. Rev. Med.*, **32**, 207.

WODE, A. (1977). In: *Martindale The Extra Pharmacopoeia*, 27th Edit., London: The Pharmaceutical Press, p. 426.

WYLLIE, A.H., KERR, J.F.R. & CURRIE, A.R. (1980). Cell death: the significance of apoptosis. *Int. Rev. Cytol.*, **68**, 251.

WYLLIE, A.H. (1980). Glucocorticoid-induced thymocyte apoptosis is associated with endogenous endonuclease activation. *Nature*, **284**, 555.